# Providing Humans With Practical, Best Practice Handling Guidelines During Human-Cat Interactions Increases Cats' Affiliative Behaviour and Reduces Aggression and Signs of Conflict

**DOI:** 10.3389/fvets.2021.714143

**Published:** 2021-07-23

**Authors:** Camilla Haywood, Lucia Ripari, Jo Puzzo, Rachel Foreman-Worsley, Lauren R. Finka

**Affiliations:** ^1^Battersea Dogs and Cat Home, London, United Kingdom; ^2^Animal, Rural and Environmental Sciences, Nottingham Trent University, Nottingham, United Kingdom

**Keywords:** human animal interactions, animal assisted interventions, cattery management, petting, gentling, felis silvestris

## Abstract

The importance of animals' experiences and associated comfort during Human-Animal Interactions (HAI), and particularly Animal Assisted Interventions (AAI), are increasingly recognised. However, there remains a paucity of published research, particularly concerning less formal but frequent HAIs to which companion animals are typically exposed, such as stroking or petting. Additionally, few practical evidence-based guides to facilitate humans' optimal animal handling and interaction in these contexts exist. A simple set of Human-Cat Interaction (HCI) guidelines were therefore created, with the aim to enhance domestic cats' comfort during generic HCI contexts. Based around a “CAT” acronym, guidelines focused on providing the cat with choice and control (“C”), paying attention (“A”) to the cats' behaviour and body language and limiting touch (“T”), primarily to their temporal regions. Guidelines were presented to human participants during a brief training intervention, and guideline efficacy was subsequently assessed. Domestic cats available for rehoming at Battersea Dogs and Cats Home, UK (*n* = 100) were filmed during interactions with novel members of the public (*n* = 120). Cats were exposed to a maximum of six, 5-min interaction sessions, balanced across “control” (interactions with humans pre-training) and “intervention” conditions (interactions with humans post-training). For each observation, cat behaviour and posture were coded and humans' cat-directed behaviour rated on the degree to which it reflected best practice principles. Data were extracted from a total of 535 observations and average human interaction ratings and cat behaviour values compared between control and intervention conditions via paired Wilcoxon tests. Compared to the control, humans' interaction styles were rated as significantly more closely aligned with best practice principles in the intervention condition. Cats also displayed significantly greater frequencies and/or durations of affiliative and positively-valenced behaviours in the intervention. In contrast, cats in the control displayed significantly greater frequencies of human-directed aggression, in addition to greater frequencies and/or durations of behaviours associated with conflict and negative valence. Results demonstrate the positive impact of practical interaction guidelines on cats' social behaviour and comfort during HCI, with the potential to improve cats' general experiences during interactions, reduce human-directed aggression and ultimately improve cat-human relationships.

## Introduction

As scientific interest in the value of Human Animal Interactions (HAIs) grows, our understanding of their dynamics and associated impacts to both human and animal parties increases. Over the past several decades, pet ownership and various forms of HAI, including Animal Assisted Interventions (AAIs), have been investigated for their potential benefits to humans' physical and mental health, in addition to their support of children's learning, literacy and the development of prosocial behaviour [see reviews by Barker and Wolen (1), Brelsford et al. (2)]. In this regard, however, the general body of literature remains largely anthropocentric, with considerations for animals' perspectives in HAI contexts gaining traction comparatively recently [e.g., (3, 4)]. Historically, investigation into the impacts of HAIs on animals has been limited to agricultural contexts, with a heavy emphasis on animal productivity and predominantly negative aspects of wellbeing, such as stress, fear and anxiety [e.g., (5–7)]. Few studies have sought to investigate the benefits of HAIs for animals or explored the impact of HAIs on companion populations.

Despite their limited representation within welfare-based HAI literature, companion animals' social significance within human society [e.g., (8, 9)] means their interactions with humans likely represent a substantial portion of all HAIs taking place. For species such as domestic cats, the majority of these HAIs likely occur with their caregivers in the domestic home or animal rescue/rehoming environment. During HCIs, cats can be observed displaying a range of affiliative behaviours (e.g., a vertically raised tail on approach, purring, kneading and rubbing against the person), which are generally assumed to be indications of their enjoyment of, and willingness to participate in, HCI (10–12). In both home and rehoming centre contexts, cats may also show preferences for human interaction over food and toys (13), suggesting the potential value of HCI to individuals.

However, it is not appropriate to assume that HCIs are always of mutual benefit to both parties. For example, the relatively high occurrence of cat human-directed aggression amongst cat-owning households (14–16) is potentially indicative of cats' discomfort during HCI. In a large survey of Brazilian cat owners (15), cat aggression was reported by almost 50% of respondents and was most likely to occur in situations where owners were directly interacting with their cats in a social context (such as during petting or play). Aggressive responses were also more likely to occur amongst cats described as “disliking” petting. At the same time, a lack of aggressive response does not necessarily imply enjoyment, or an absence of negative experience for the cat, as significantly higher faecal cortisol metabolite levels were found amongst cats that were described as “tolerating” rather than actively “liking” or “disliking” being stroked (17).

Cats' desire for, and experiences during HCI are likely to be context dependent and moderated by their individual characteristics (such as temperament), in addition to the behaviour and characteristics of the human. For example, in novel environments or stressful situations, cats tend to seek out physical places of safety and security (18), and even otherwise friendly cats may prioritise these resources over social interactions with humans, particularly during periods of habituation. In a shelter environment, both fearful and frustrated cats may benefit from regular HCI, but only if humans are perceived as a positive (non-threatening) stimulus [e.g., (19, 20)]. Otherwise, human proximity and associated HCI may have negative, or at the least less positive, impacts on well-being [e.g., (19, 21)], particularly if the cat does not have the option to effectively “opt in” or “opt out” of the HCI [e.g., (19)].

Cats are also likely to value choice and control during HCI and to prefer humans that are sensitive to their behavioural responses and associated needs. Amongst well-socialised cats, individuals tend to prefer to interact with humans that do not approach them when they are resting, nor follow them when they are attempting to retreat, but instead adopt a lowered (cat height) position and vocalise to them [e.g., (22)]. Cats also tend to prefer HCI that they themselves initiate, and will respond more positively to humans that are generally more responsive to their requests for interaction (23). Finally, cats appear to have preferences for the regions of their bodies that are touched during HCI. Stimulation of cats' temporal regions is likely to induce more positive responses, whilst stimulation of the caudal region may have the opposite effect (11, 24). In contrast, stimulation to the cats' perioral, flank, stomach, and back areas may show more varied responses, depending on the their individual preference (11). The provision of sufficient autonomy and the importance of observing animals' reactions during HAI are thus fundamental to ensuring relationships with humans have a positive impact on animals (25).

With the growing popularity of cats being included in HAI outside of the domestic home, cats are being exposed to HCI across increasingly diverse landscapes, in novel environments, with novel people. For example, the involvement of cats within both educational and therapeutic forms of AAI appears to be on the rise. These range from cats being placed with families to provide social and emotional support to children diagnosed with Autism Spectrum Disorder (26), to cats housed in shelters being visited and read to by children [e.g., (27)], and cats visiting care facilities to provide emotional and physical health benefits to the ill and elderly (28, 29). Initially a Japanese phenomenon (30), the growing international popularity of Cat Cafés present an additional (non-interventional) context, where cats are exposed to HCI for humans' benefit. Concerns over the negative impacts of Cat Cafés to cats (e.g., due to their being constantly handled by unfamiliar humans) have been raised (31, 32). Similar sentiments are also increasingly echoed in relation to the broader welfare implications of the inclusion of both companion and non-companion animals in HAIs such as AAI (33, 34), but also animal-based tourism (32).

To ensure HCI are enjoyable for cats, an understanding of their desire for, and preferences during, HCI are crucial. Evidence-based guidelines that translate these preferences into practical, species-specific and easy to implement actions are however missing, particularly outside of formal veterinary handling contexts [e.g., (35, 36)]. The development of simple, generic cat-interaction guidelines could therefore be extremely useful in supporting cats' well-being and enjoyment during all forms of HCI, including those that occur in the domestic home and rehoming centres, but also those taking place in interventional (i.e., AAI) and other tourism based contexts (e.g., Cat Cafes).

The purpose of the current study was to therefore test the efficacy of a set of “best practice” informed human cat-interaction guidelines, when introduced to humans during a brief training intervention. Efficacy was primarily determined via the objective quantification of cats' behavioural responses during HCI, focusing on their human-directed social behaviour (both agonistic and affiliative), as well as more general indicators of comfort (e.g., behaviours linked to positive and negative affect and/or conflict). To maximise general efficacy, the guidelines were designed to be sufficiently generic to enable cross-context application and to be usable by individuals without professional knowledge of cat behaviour. For this study, we therefore assessed guideline efficacy when applied by general members of the public within a rehoming centre context. This provided the additional benefit of easily controlling for human-familiarity and environmental effects during HCI. Given that cats' temperament can differentially mediate the well-being impact of HCI in shelter settings (19), we also sought to determine whether individuals might benefit more or less from humans' implementation of the guidelines, depending on the cats' temperament. For example, cats with emotional predispositions toward anxiety and/or frustration during HCI, might be predicted to respond relatively more positively (or at least less negatively) when humans followed the guidelines. In contrast, highly gregarious cats might be less sensitive to differences in humans' handling styles and therefore show little difference between conditions.

Overall, the guidelines encouraged a more hands-off or restrictive approach than most people might prefer when petting cats. In the rehoming context, the initial HCI between prospective adopters and cats are likely crucial to peoples' decision making. Therefore, to ensure that the guidelines could be effectively applied in this context without negatively affecting rehoming rates, we also sought to assess their effect on peoples' perceptions of individual cats during HCIs.

Specifically, our aims were to:

i) Determine whether the training intervention had a positive impact on the general handling styles cats were exposed to (i.e., did intervention handling align more closely to best practice principles, compared to the control?)
ii) Determine whether the training intervention had a positive impact on the behavioural responses of cats during HCI (i.e., did cats respond more positively and less negatively toward participants in the intervention condition?)
iii) Determine whether the temperament of the cat moderated the impact of the intervention on their behavioural responses (i.e., depending on their temperament, did some cats display relatively more positive and less negative behaviours in the intervention condition?)
iv) Determine whether adherence to a potentially more restrictive form of HCI might negatively impact humans' impressions of cats (i.e., did participants rate cats less positively during the intervention condition?).

## Methods

All supplementary materials are available via https://doi.org//10.6084/m9.figshare.14828397.

### Development of the “CAT” Interaction Guidelines

The interaction guidelines were developed by author LF and aimed to reflect current best practice methods of interactions with cats, informed by a combination of expert opinion (e.g., LF and colleagues) and the (limited) published evidence on this topic [e.g., (11, 22–24)]. Based around a “CAT” acronym, the guidelines aimed to provide a memorable and easy to implement set of instructions for people to follow during all HCI contexts, with the exception of situations requiring specific handling for formal veterinary procedures or specific husbandry activities. The “C” represented providing the cat with Choice and Control during the HCI, enabling them to both “opt in” and “opt out.” The “A” encouraged people to pay attention to the cats' behavioural and postural responses during interactions, and to moderate their behaviour accordingly. The “T” encouraged people to restrict their touching of cats primarily to the cats' temporal regions (see Table 1).

**Table 1:** A summary of the key principles of the “CAT” interaction guidelines explained to participants during the training intervention.

C choice and control	**Provide the cat with choice and control during the interaction**
	• While remaining in your seated position, gently offer your hand to the cat, allow the cat to approach you, and let them choose if they want to interact with you or not • If the cat wants to be touched, he or she will rub against you. If they don't make contact, avoid stroking the cat • Allow the cat to move away from you if they choose, and don't be tempted to follow after them • Allow the cat to control how much you stroke them. If stroking the cat, briefly pause every 3–5 s to “check in” with the cat–when you stop stroking them, do they rub against you to ask for more? If not, they may be ready for a break
A attention	**Pay attention to the cat's behaviour and body language**
	The following are signs that the cat may need a little break: • The cat turns it head or moves away from you • Their ears become flattened or rotate backwards • They shake their head • The fur on their back appears to ripple • They lick their nose • They go a bit still, and stop purring or rubbing against you • They sharply turn their head to face you or your hand • They suddenly start grooming themselves, lasting only a few seconds • Their tail twitches or ‘swishes' vigorously, usually when held horizontally or close to the ground
T touch	**Think about where you're touching the cat**
	• Most friendly cats will prefer being touched at the base of their ears, around their cheeks, and some also under their chin, so try to stick mainly to these areas • Avoid the base of their tail and tummy, and be cautious then touching the cat's back, flank, legs, and tail–pay close attention to their body language to see if they appear comfortable

### Data Collection

Data collection took place at Battersea Dogs and Cats Home, Battersea, London, UK between 20th January and 13th March 2020 and was carried out by authors LF, RFW, CH and JP. Participants were recruited on a voluntary basis via an online advert circulated on social media, with a similar version also sent directly to Battersea's LinkedIn contacts. Participant contact details were collected for the purposes of arranging testing slots only. Upon arrival, each person was allocated a reference number so that the subsequent study data collected could be fully anonymised. Participants were given a short verbal introduction to the study but were not told of the specific study aims (i.e., to investigate the impact of the training intervention on cats' behaviour). Participants were asked to complete a short survey that included very basic demographic questions (i.e., age and gender), in addition to several questions about cat ownership and experiences with cats. The second part of the questionnaire included the 44-item Big Five Inventory (BFI) to assess human personality (37) (see Supplementary File 1 for a copy of participant questionnaire). Data extracted from this questionnaire is currently being analysed for inclusion in further publications. Participants visited six different cats, three prior to and three after receiving training on the “CAT” guidelines. These conditions reflected the “control,” and the “intervention” conditions, respectively.

### Cat and Human Demographics

A total of 114 cats were initially included in the study. Almost all cats (93%) were neutered at the time of testing. Forty two percentage were male and 58% were female, with an average age of 6.1 years (sd 4.3 years). With the exception of 4 cats (a British short hair, Somali, Burmese, and Ragdoll) all study cats were domestic short or semi-long haired (see Supplementary Data 2 for full details).

A total of 120 participants took part in the study, the vast majority of which were female (90%). Ages spanned the following ranges, 18–25 (9%), 26–35 (30%), 36–45 (25%), 46–55 (21%), 56–65 (12%), and 66–75 (3%), and 57% of participants currently lived with at least one cat. To reduce the collection of unnecessary sensitive data, no further demographic information were collected from participants.

### Test Protocol

#### Control Condition

Participants visited three different cats for 5 min each. They were initially presented with a brief instruction sheet explaining the protocol (Supplementary File 3) before being instructed to quietly enter the cats' pen and to then sit in the corner nearest to the door, facing diagonally toward the back corner. Optimal camera placement and participant positioning was determined during piloting. Two GoPro HERO7 cameras were subsequently mounted on flexible mini tripods and attached (roughly 1–1.5 m from the ground) to the front and back sections of each cats' pen, facing inwards and angled downwards, in order to capture the whole area of the pen. The control condition was designed to encourage relatively “naturalistic” interactions between humans and cats, whilst ensuring the cat was protected from handling that might cause them distress or lead to participant injury. Participants were therefore instructed to interact with the cat as they usually would, without picking the cat up or restraining them, and to remain in their seated position for the duration of the test. This ensured that if the cat chose to retreat or hide during the HCI, they could do so without the risk of being disturbed. To reduce external visual and acoustic disturbance during tests, sessions predominantly took place on the cattery floors that were off-access to the general public. In the rare cases where cats located on the public floor were used, this occurred during quieter periods of the day. Once the test began, a dark curtain was placed over the door of the cats' pen to reduce the impact of external disturbance. For infection control purposes and to remove the scent of previous cats, participants were instructed to sanitise their hands with Anigene (Medimark Scientific) hand sanitiser in between cats.

#### Intervention Condition

Following the control condition, participants were exposed to a short training intervention. This consisted of a 5-min educational video created by LF (Supplementary Video 4), explaining and visually demonstrating the CAT guidelines (Table 1) whilst a cat was present. Participants were then presented with an instruction sheet (Supplementary File 5) that further highlighted key points of the CAT guidelines. Following the training, participants visited three additional cats using a similar test protocol to the control condition, with the expectation that they were requested to follow the CAT guidelines. As a further prompt, and to encourage compliance during HCI, a laminated poster containing the CAT acronym was attached to the wall in the cats' pen during each test (Supplementary File 6).

#### Experimental Set Up

Each cat was housed singly in a pen measuring approximately 2 × 3 × 1.5 m. All cats were provided with a litter tray, several concealed areas (one elevated and another at ground level, located in the back section of their pen), blankets, toys, a scratching post, and water. Cats were fed and provided with a clean litter tray twice daily. Cleaning, feeding and opportunities for human interaction followed a predictable daily schedule. On test days, cats were not provided with opportunities for human interaction outside of those occurring during feeding and cleaning. This was to standardise the amount of social interaction to which test cats were exposed and to avoid possible carry over effects from interactions with staff or volunteers prior to testing.

#### Participant Inclusion Criteria

Participants were required to be aged 18 or over, comfortable interacting with cats whilst sitting or kneeling on the floor for short periods of time and also willing to travel to Battersea on an agreed date and time.

#### Cat Inclusion/Exclusion Criteria

Cats were required to be >6 months of age and physically healthy (i.e., not currently in pain or experiencing any acute health complaints). At the time of testing, cats needed to have been occupying their respective pen in the cattery for a minimum of 48 h to support initial habituation/acclimatisation to the cattery environment [e.g., (38, 39)]. Cats deemed notably stressed, unsettled or uncomfortable were not enrolled in the study on welfare grounds, and to ensure sufficient HCI data could be collected (given such cats would likely remain hiding for the duration of the test, irrespective of human interaction style). For inclusion, cats had to be deemed well-socialised to humans and considered suitable to be rehomed to live with humans as a companion (i.e., rather than requiring a “non-pet” outlet such as a farm).

#### Cat Testing Order

All cats were tested between 9 a.m. and 3 p.m. to avoid feeding and cleaning times. Cats were tested in blocks of 12, over the course of two consecutive days, receiving 6 tests in total and 3 per day, each time with a novel participant. As time of day could potentially impact on the cats' behavioural responses during HCI, and the number of previous cats a person had visited (i.e., 0 compared to 5) might impact on the participants' behaviour toward a cat, these factors were controlled via a complete balanced block design [e.g., (40)]. Additionally, condition order always alternated between a “control” and an “intervention” with a minimum break of 1.5 h between each test per cat to control for potential carry over effects between tests. To provide sufficient numbers of cats for each block of testing, 17 cats were exposed to a second set of 6 tests, with a minimum break of 1 week in between testing blocks.

#### Cat Ratings

After visiting each cat, participants were asked to complete a form (see Supplementary File 7) where they rated each cat on a 5-point Likert scale for (i) how friendly and (ii) how comfortable they found the cat, in addition to (iii) how likely they would be to choose that cat if they were considering rehoming one.

#### Cat Temperament Assessment

To determine whether cat temperament might mitigate or mediate any impact of the CAT intervention on cats' behavioural responses, cattery staff filled out an L-CAT questionnaire for each cat enrolled in the study (Supplementary File 8). The L-CAT is a validated (i.e., demonstrated convergent, discriminant, and predictive validity), reliable (i.e., demonstrated inter, intra-rater and temporal stability) and practical tool which provides cats with three scores based on their perceived level of friendliness, fearfulness, and tendency toward frustration in the context of HCI (41).

### Behaviour Coding

#### Human Behaviour

To ensure that participants' interactions with cats changed in line with the “CAT” guidelines following the training intervention, a simple human handling score was assigned to each participant for each observation. The score reflected the degree to which the participant was judged to be interacting with the cat in a way that aligned with the best practice principles of the “CAT” guidelines (3 = closely, 2= somewhat, 1 = not at all).

#### Cat Behaviour

Videos were divided between authors CH and LR. Forty seven aspects of cats' behaviour were coded across all videos in BORIS coding software v. 7.9.8. (42), using a specially developed and thoroughly piloted ethogram (see Supplementary Data 2 for the full list of behaviours and their operational definitions). The ethogram was informed by previously published work and was designed to capture a range of practically codeable and easily standardised measures, typically associated with either positive or negative valence in domestic cats in social contexts [e.g., (11, 24, 36, 43–45)]. These included human-directed social behaviours (both affiliative and agonistic), in addition to relevant postural and behavioural indicators of comfort (e.g., behaviours associated with negative arousal, relaxation and positive arousal). The ethogram also included two codes (Zones 1 and 2) that were used to quantify the position of the cat relative to the human. Zone 1 represented the first third of the pen where the cat was mostly within touching distance of the participant. Zone 2 represented the rest of the pen, furthest away from the participant where the cats' main hiding and sleeping areas were located. Zones could be easily visually discriminated for standardisation, due to three equally sized glass panels positioned along the length of the pens. Duration of time the cats' head, tail, and body could not be coded (due to limited visibility) were also measured and later used to transform relevant behaviour measures into proportion data (see further). Depending on the specific nature of the behaviour, behaviours were coded as frequencies (e.g., approaches person), durations (e.g., crouch/tense posture) or as both frequencies and durations (e.g., tail wave) (see Supplementary Data 2 for ethogram details). Video eligibility for coding required the cat to be visible for a total of at least 2 min out of the 5-min test duration and to have at least one observation for both the control and intervention conditions. With the exception of the videos coded by a second coder for inter observer reliability (see further), the majority of videos could not be blind coded for several reasons. The primary coder (CH) was involved in the data collection process and was therefore aware of cat testing orders. Additionally, the CAT guidelines attached to the wall during the intervention condition (to act as a “prompt” for participants) were clearly visible within most videos, and therefore this condition was easily identifiable to those familiar with the test protocol (e.g., CH).

#### Inter-rater Reliability Coding

A sample of 20 videos coded by CH were pseudo-randomly selected for inter-rater reliability coding, ensuring an equal number of “control” and “intervention” conditions were included, and that each video was of a different cat. Selected videos were blind coded by LR (who was unfamiliar with the test protocol).

### Data Preparation

#### Collapsing of Measures With Low Occurrences

To provide a more detailed, exploratory picture of potential differences in cats' behavioural responses between conditions, we opted to avoid the collapsing/grouping of behaviour variables prior to analysis where possible. However, due to the relatively low individual occurrence of frequency-based behaviours linked to conflict/negative affect (*n* = 6, see ethogram in Supplementary Data 2) within both conditions, it was necessary to collapse these variables into a “conflict” composite score, so that their values could be analysed statistically. For the same reasons, this process was also undertaken for frequency-based measures relevant to agonistic behaviour (*n* = 3), creating an “agonistic” composite score (see Supplementary Data 2 for further details of measures). A total of 40 cat behavioural measures were therefore assessed, including their inter-rater reliability, in addition to the human-handling ratings.

#### Creation of Averaged Measures for Each Condition

To account for potential inter-individual variation in human handling and human perceptions of cats within conditions, single averaged scores were generated for each cat for human-handling and human cat-ratings, in both the control and intervention conditions.

Behaviour measures retained post-reliability analysis (see further), were transformed into proportion data based on the duration of time each measure could be coded within each video due to the cats' visibility. Average proportion values were then generated for each cat for both the control and intervention conditions (see Supplementary Data 2 for full dataset).

#### Cats With Missing Data

##### Excluded Cats

Of the 114 cats initially included in the study, 14 were subsequently excluded from the dataset due to (i) being visible for <120 s for each of their observations (*n* = 6), or (ii) not having at least one observation from both the control and intervention condition. The latter occurred due to early removal from the study for health and welfare reasons (*n* = 4), the cat being rehomed (*n* = 2) and concerns for participants' safety (*n* = 2).

##### Included Cats

From the remaining cats (*n* = 100), a total of 586 videos were coded. Of these 100 cats, 30 did not have a full set of 6 observations, due to video camera malfunction (*n* = 5), early removal from the study due to rehoming (*n* = 5), health or welfare reasons (*n* = 3), or the cat being visible for <120 s during some of their observations (*n* = 22). To retain as large a dataset as possible, in these instances, averaged values for the control and intervention conditions were created from 2 rather than 3 observations (*n* = 19 for control, *n* = 19 for intervention), or a single non-averaged value was used (*n* = 10 for control, *n* = 7 for intervention). In the cases where cats had been exposed to two blocks of tests (i.e., 2 × 6 observations, *n* = 17), observations from their first block of 6 tests were extracted. If there were any missing observations from the first block, data were supplemented from their second block, in order to create a full set of 6 observations. Of the initial total of 586 observations coded, data were extracted from 535.

### Statistical Analysis

All statistical analyses were undertaken in R version 4.0.2 (46) using functions within the “psych” package (47), “base” and “stats” packages (46). Boxplots were generated via “ggplot2” (48).

#### Inter-rater Reliability

Inter-rater reliability for both the behaviour measures and human-handling scores were assessed via Intraclass Correlation Coefficients (ICC2), a measure of absolute agreement between raters (49), with an ICC2 threshold of <0.5 used to identify measures with poor agreement (50). Measures with poor agreement (*n* = 3, see Supplementary Data 2) were excluded from subsequent analyses.

#### Differences Between the Control and Post-intervention Condition

Differences in average control and intervention scores for all the behaviour measures as well as “human-interaction” and “cat ratings” were non-normally distributed (Shapiro-Wilk tests, *p* < 0.05) and thus analysed via paired Wilcoxon signed rank tests. All behaviour measures with acceptable levels of inter-rater reliability were analysed (*n* = 31), with the exception of the frequency and duration values (*n* = 6) that were used to calculate relative proportions for the other measures (i.e., head/body/tail not visible).

Due to the exploratory nature of this study and the lack of “gold standard” measures of behaviour when testing the effect of an intervention of this nature on cats' behavioural responses, we opted to test differences in behavioural outcomes across a range of individual measures that were considered context appropriate and of high biological relevance. To avoid the risk of type 2 errors, we opted against performing any power reducing corrections (e.g., Bonferroni) and instead chose an alpha value of *p* < 0.05 to determine significance and also calculated the effect size for each measure [as recommended by Nakagawa (51)], using the standard formula for non-parametric data (*r* = Z/√N). The r value varies from 0 to close to 1, with values of 0.10 to <0.3 considered indicative of small effects, 0.30 to <0.5 moderate effects and >0.5 large effects (52).

##### Data Visualisation

Data were plotted via a series of boxplots in order to visualise the relative difference in values between the pre and post-intervention conditions.

#### Interactions Between Cat Temperament and Relative Difference in Behaviour Between Conditions

Outcomes of the Wilcoxon tests highlighted several measures associated with affiliative behaviour and/or positive affect that were significantly greater in the intervention condition (*n* = 5, see results for full details), as well several measures associated with social discomfort/negative affect (*n* = 4) that were significantly greater in the control. These respective “positive” and “negative” affect linked measures were subsequently summed to create “positive” and “negative” composite scores for each cat for both the control and intervention conditions. Both frequency and duration values were included in each composite score, thus all measures were scaled (using the “scale” function in r) prior to summing. Relative differences in “positive” and “negative” scores between the two conditions were then calculated by subtracting the respective intervention score from the control. Separate Generalised Linear Models (GLM) were performed, with either the “relative positive” or “relative negative” composite score included as the response variable and the three temperament scores and their interaction as the explanatory variables (i.e., frustration^*^friendliness^*^fearfulness). A summary of the full model was called to identify potential effects of the explanatory variables. In both cases, all explanatory variables and their interactions were non-significant, thus a lack of effect was then confirmed by comparing each full model to the null model via ANOVA chi-square tests. Five cats were missing L-CAT scores, therefore data used in the GLMs was n = 95.

## Results

### Inter-rater Reliability

With the exception of three measures relating to treading frequency, crouching duration and tail parallel duration, reliability coefficients for the behaviour measures and human handling score were generally well-above the acceptability threshold of 0.5. ICC values ranged from 0.64 to 1 (see Supplementary Data 2).

### Differences Between the Pre and Post-intervention Conditions

#### Human-Interaction Scores

**Figure 1:**
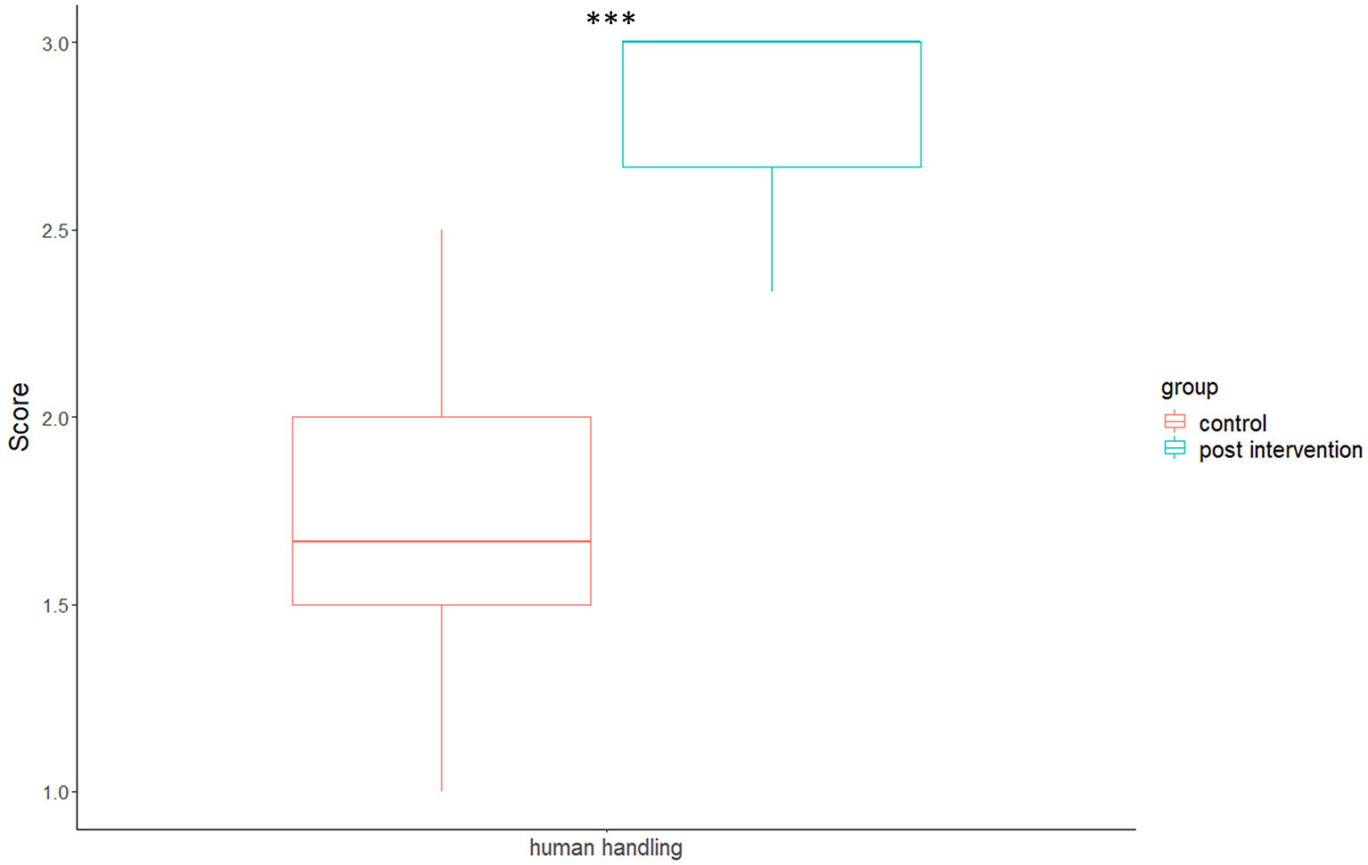
Boxplots of humans' handling scores for control and intervention conditions (*p < 0.05, **p < 0.01, ***p < 0.001).

Average human-handling scores were significantly higher (indicating greater compliance with best practice) in the intervention compared to control condition [*p* < 0.001, *v* = 8.5, *r* = 0.866, mean (control) score = 1.7189, mean (intervention) score =2.803], see Table 2 and Figure 1.

**Table 2:** Paired Wilcox test outputs and descriptive statistics of all behaviour measures coded across the control and intervention conditions for *n* = 100 cats.

**Measures**	**Paired wilcox test values**			**Descriptive statistics–control**				**Descriptive statistics–intervention**			
	***P*-value**	***V*-value**	**Effect size (r)**	**Mean**	**sd**	**Median**	**se**	**Mean**	**sd**	**Median**	**se**
Zone1_dur	0.8004	2,252	0.0120	0.8688	0.1602	0.9395	0.0160	0.8486	0.2064	0.9505	0.0206
**Zone1_freq**	**0.005102**	**3,340**	**0.280**	**0.0068**	**0.0049**	**0.0051**	**0.005**	**0.0056**	**0.0035**	**0.0044**	**0.0003**
Zone2_dur	0.7768	2,111	0.0148	0.1307	0.1605	0.0588	0.0161	0.1514	0.2064	0.0495	0.0206
**Zone2_freq**	**0.002631**	**2,971**	**0.307**	**0.0067**	**0.0067**	**0.0051**	**0.0007**	**0.0051**	**0.0050**	**0.0033**	**0.0005**
**Tail_s_dur**	**0.0142**	**3,118**	**0.245**	**0.1752**	**0.1778**	**0.1188**	**0.0178**	**0.1487**	**0.1601**	**0.0860**	**0.0160**
Tail_s_freq	0.2188	2,773	0.126	0.0257	0.0349	0.0178	0.0035	0.0223	0.0193	0.0167	0.0019
**Tail_w_dur**	**0.0001388**	**775**	**0.379**	**0.0117**	**0.0359**	**0.0021**	**0.0036**	**0.0160**	**0.0215**	**0.0089**	**0.0022**
**Tail_w_freq**	**3.564e-05**	710	**0.416**	**0.0017**	**0.0025**	**0.0011**	**0.0003**	**0.0032**	**0.0036**	**0.0022**	**0.0004**
**Ears_f_dur**	**4.256e-06**	1,157	**0.459**	**0.7297**	**0.2207**	**0.7877**	**0.0221**	**0.8120**	**0.1599**	**0.8440**	**0.0160**
Ears_f_freq	0.3162	2,817	0.100	0.0191	0.0096	0.0182	0.0010	0.0186	0.0091	0.0178	0.0009
**Ears_r_dur**	**1.264e-08**	**4,106**	**0.569**	**0.2636**	**0.2184**	**0.2085**	**0.0218**	**0.1713**	**0.1435**	**0.1267**	**0.0144**
**Ears_r_freq**	**0.02019**	**3,141**	**0.233**	**0.0166**	**0.0097**	**0.0156**	**0.0010**	**0.0151**	**0.0090**	**0.0145**	**0.0009**
Roll_dur	0.9527	1,129	0.00929	0.0813	0.1202	0.0172	0.0120	0.0900	0.1557	0.0050	0.0156
Roll_freq	0.3131	1,301	0.0625	0.0037	0.0056	0.0011	0.0006	0.0031	0.0053	0.0011	0.0005
**Sniff_dur**	**0.04881**	**1,869**	**0.196**	**0.0405**	**0.0636**	**0.0140**	**0.0064**	**0.0467**	**0.6072**	**0.0231**	**0.0067**
Sniff_freq	0.1232	1,990	0.153	0.0098	0.0096	0.0068	0.0010	0.0113	0.0094	0.0091	0.0009
Approach	0.6786	2,404	0.0416	0.0109	0.0077	0.0090	0.0008	0.0110	0.0069	0.0089	0.0007
Meow	0.9146	507	0.0556	0.0040	0.0143	0.0000	0.0014	0.0034	0.0104	0.0000	0.0010
**Tread_dur**	**3.394e-06**	**255**	**0.470**	**0.0963**	**0.2325**	**0.0000**	**0.0232**	**0.1394**	**0.2654**	**0.0082**	**0.0265**
**Phys_cont_dur**	**0.001684**	**3,439**	**0.314**	**0.6795**	**0.2244**	**0.7098**	**0.0224**	**0.6057**	**0.2516**	**0.6167**	**0.0252**
Phys_cont_freq	0.3453	2,800	0.0946	0.0371	0.0386	0.0318	0.0039	0.0335	0.0215	0.0300	0.0022
**Rub**	**1.396e-14**	**286**	**0.770**	**0.0450**	**0.0334**	**0.0385**	**0.0033**	**0.0801**	**0.0464**	**0.0782**	**0.0046**
**Confl_disc**	**0.0006854**	**3,513**	**0.340**	**0.0189**	**0.0189**	**0.0156**	**0.0018**	**0.0138**	**0.0087**	**0.0121**	**0.0009**
**Agonistic**	**9.111e-05**	**500**	**0.380**	**0.0017**	**0.0041**	**0.0000**	**0.0004**	**0.0005**	**0.0014**	**0.0000**	**0.0001**
Tail_u_dur	0.2935	2,831	0.105	0.4333	0.2500	0.4191	0.0250	0.4223	0.2365	0.4187	0.0236
Tail_u_freq	0.8784	2,570	0.0155	0.0194	0.0106	0.0179	0.0011	0.0194	0.0108	0.0189	0.0011
Tail_p_freq	0.137	2,092	0.149	0.0136	0.0111	0.0111	0.0011	0.0146	0.0110	0.0122	0.0011
Tail_d_dur	0.488	1,551	0.0705	0.0470	0.0866	0.0118	0.0089	0.0550	0.0936	0.0144	0.0094
Tail_d_freq	0.9547	1,756	0.0102	0.0063	0.0110	0.0022	0.0011	0.0054	0.0084	0.0022	0.0008
Tail_o_dur	0.2832	2,783	0.107	0.3713	0.2528	0.3648	0.0253	0.3554	0.2406	0.3177	0.0241
Tail_o_freq	0.5017	2,282	0.0683	0.0139	0.0099	0.0111	0.0010	0.0140	0.0100	0.0122	0.0010
**Human handling (scale** **=** **1–3)**	** <2.2e-16**	**8.5**	**0.866**	**1.7189**	**0.4108**	**1.6667**	**0.0413**	**2.803**	**0.2987**	**3**	**0.0300**
**Human cat rating (scale** **=** **1–5) “Friendly”**	0.2386	1,606	0.0817	4.3201	0.7461	4.6667	0.0702	4.2832	0.7292	4.3333	0.0686
“Comfortable”	0.7425	1,590.5	0.0235	4.3201	0.6543	4.3333	0.0616	4.3422	0.6751	4.5000	0.0635
“Rehomable”	0.2678	1,536.5	0.0913	4.1504	0.7539	4.3333	0.0709	4.1976	0.7597	4.3333	0.0715
**L-cat personality**	Maximum	Minimum	Mode	Mean	sd	Median					
“Friendliness” (scale = 4–20)	20	11	19	16.21296296	2.467028	17					
“Fearfulness” (scale = 3–15)	13	3	9	7.486238532	2.730607	8					
“Frustration” (scale = 3–15)	9	3	3	3.42201835	1.450801	3					

*Measures with significant differences (p <0.05) between conditions are indicated in bold*.

#### Human Cat-Behaviour Ratings

Average “friendly,” “comfortable,” and “rehomeable” ratings given to cats by participants did not differ significantly between the control and intervention conditions (all *p* > 0.05, Table 2).

#### Cat Behaviour Measures

**Figure 2:**
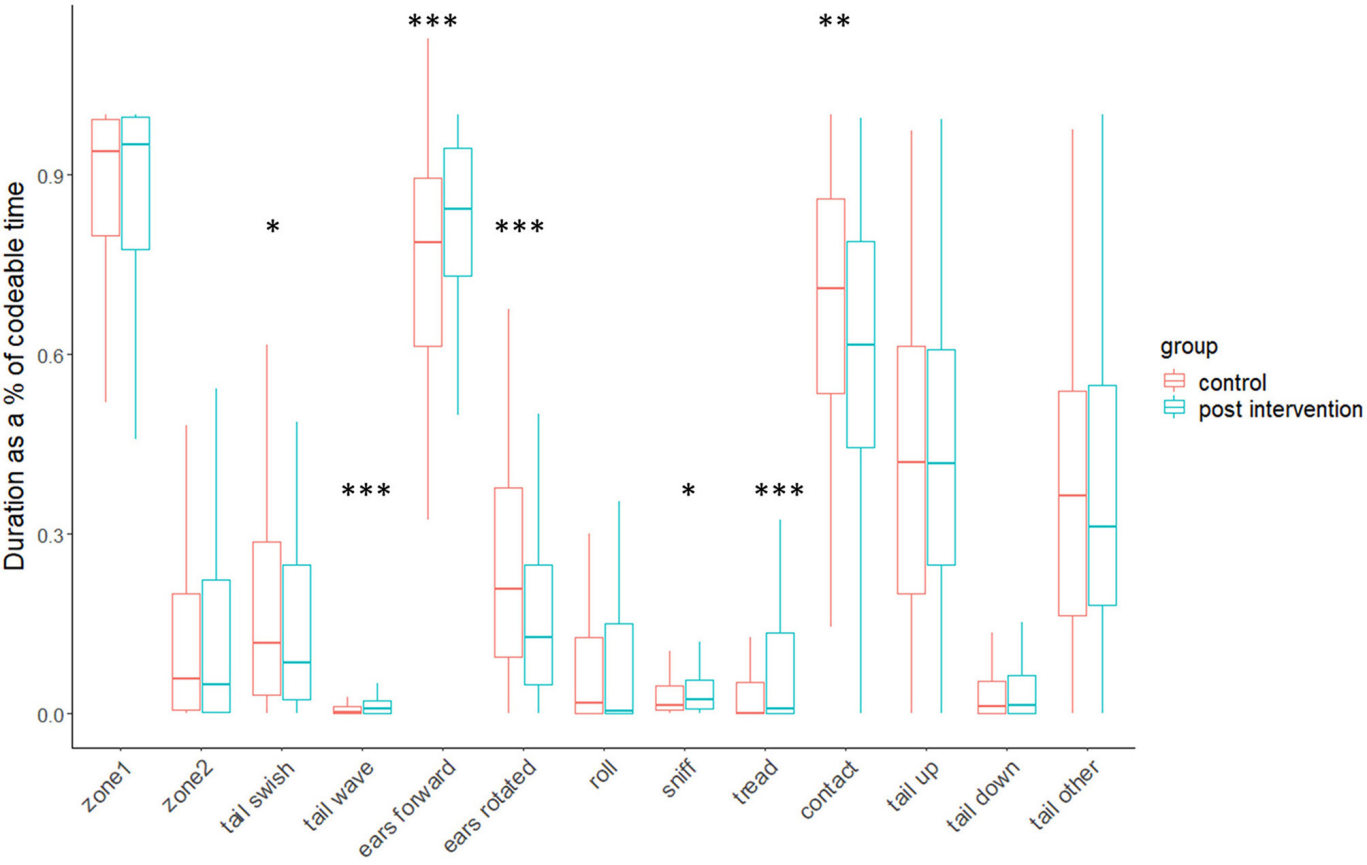
Boxplots of cats' average (duration based) behavioural responses for control and intervention conditions (*p < 0.05, **p < 0.01, ***p < 0.001).

**Figure 3:**
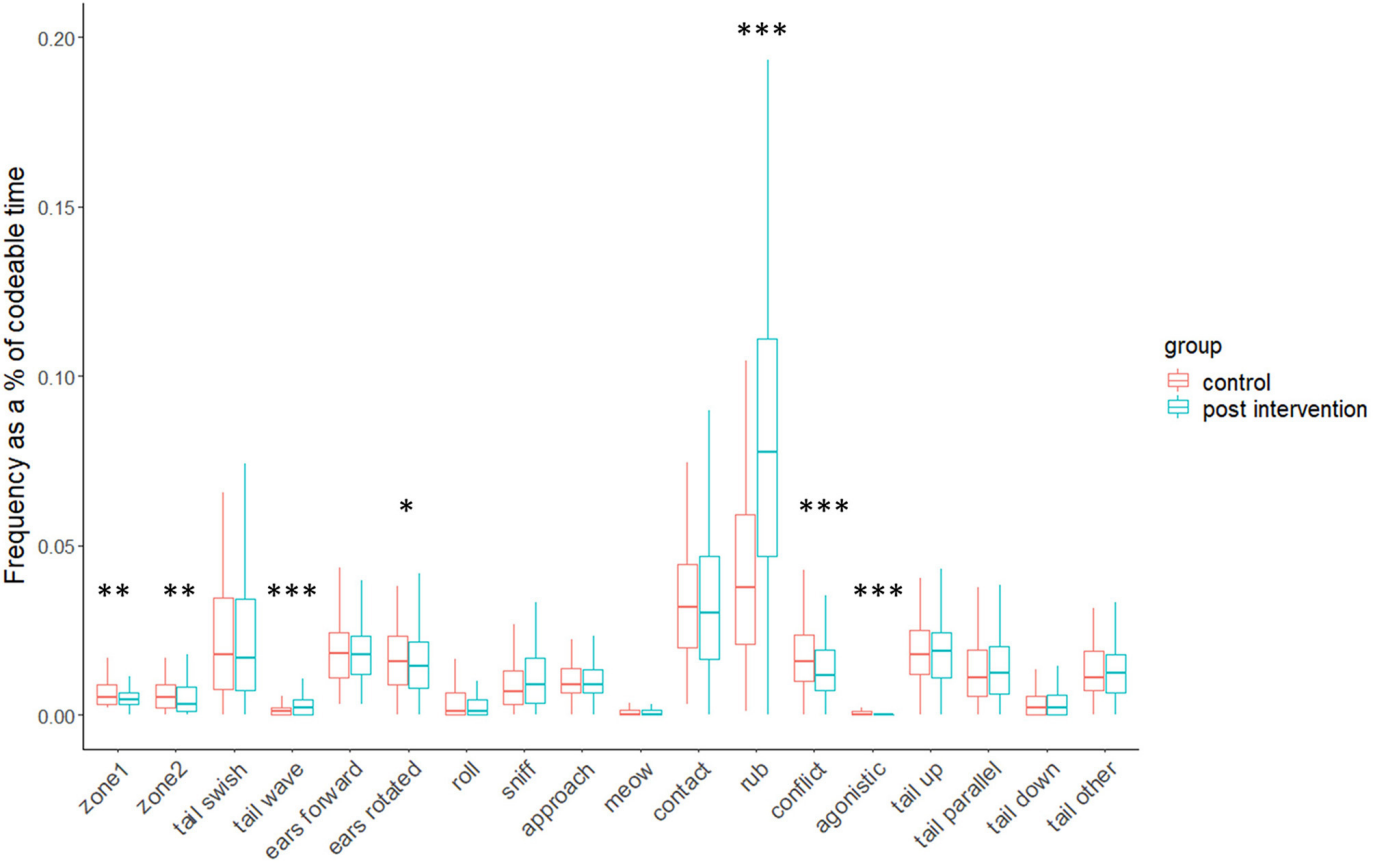
Boxplots of cats' average (frequency based) behavioural responses for control and intervention conditions (*p < 0.05, **p < 0.01, ***p < 0.001).

Various frequency and duration-based measures differed significantly (*p*-values ranged from *p* < 0.001 to *p* < 0.05) between the control and intervention conditions (for full results, see Table 2 and Figures 2, 3). Associated effect sizes were generally moderate (i.e., 0.30 to <0.5). The exceptions included small effects (i.e., 0.10 to <0.3) for frequency of entering zone 1, frequency of ears rotated and/or flattened, and durations of sniff person and tail swish, and large effects (i.e., >0.5) for duration of ears rotated and/or flattened, frequency of rub/paw person, and also human-handling score.

#### Summary of Differences in Affiliative and Positive Affect-Linked Behaviours

Compared to the control, on average, cats in the intervention condition waved their tails for significantly longer and more frequently, had their ears in a neutral or forwards position for longer, “treaded” or “kneaded” with their front paws for longer, sniffed the participant for longer and also rubbed against them more frequently.

There were no significant differences in the average frequencies with which cats approached and made contact with the participants between conditions, although cats were in physical contact with participants for significantly longer average durations in the control.

#### Summary of Differences in Agonistic and Negative Affect-Linked Behaviours

Agonistic events occurred amongst 27% of cats in at least one of their observations for the control and amongst 16% of cats in at least one of their observations in the intervention condition. Average agonistic scores (i.e., composite of hiss/growl, cuff/swipe, bite) were significantly higher in the control condition, compared to the intervention.

Average conflict/negative affect scores (i.e., composite of paw lift, rapid groom, head/body shake, freeze/crouch, sharp turn of head toward participant, avoid/move/turn away from participant) were significantly higher in the control condition, compared to the intervention. On average, cats also swished their tails for significantly longer durations and rotated and/or flattened their ears more frequently and for longer durations in the control.

#### Differences in Pen Location

On average, cats changed their position between zone 1 (nearest the participant) and zone 2 (furthest away from the participant) significantly more frequently in the control condition, but did not spend significantly longer durations in either zone between conditions.

### Interactions Between Cat Temperament and Relative Differences in Control and Intervention Behaviour

Results of the GLMs indicated a lack of significant relationship between cats' temperament scores (and their interactions) and the relative difference in their “positive” and “negative” behaviour composite scores between conditions. In both cases, outputs of the model summary and subsequent comparisons of the full and null models yielded non-significant (both *p* > 0.05) values, with the null models producing the lowest Akaike information criterion (AIC) values in both cases.

## Discussion

The results of this study demonstrate the beneficial impact of a simple set of cat-interaction guidelines on cats' real time responses to humans during HCI. Humans' general interaction style was significantly more closely aligned to “best practice” principles following the training intervention. Additionally, not only did cats behave less aggressively during the intervention condition, but they also performed fewer behaviours associated with conflict or negative affect, as well as more human-directed affiliative behaviours and those associated with positive affect. Collectively, these results suggested that the guidelines are easy for non-experts to understand and implement, and may facilitate safer and more beneficial HCIs for both cats and humans.

Perhaps counterintuitively, cats were in physical contact with humans for significantly longer average durations in the control compared to the intervention condition. However, as this measure did not differentiate between whether the contact was initiated by the cat or the human, it is likely that the direction of differences in this measure are reflective of participants adopting a more “hands-off” approach following training (as encouraged by the CAT guidelines). As cats otherwise reacted more positively and less negatively in the intervention condition (where physical contact occurred for shorter periods), a “less is more” approach is likely relevant when it comes to cats' preferences for physical contact during HCIs, even though cats rubbed against participants more frequently in this condition. Durations of time spent in either Zone 1 or 2 were not significantly different between conditions, however cats entered in (and out) of both Zones significantly more frequently in the control. As both zones represented the cats' relative proximity to the participant (i.e., Zone 1 was nearest to the person and Zone 2 furthest away), such behaviours are potentially indicative of greater participant-directed distance increasing/decreasing behaviour in the control, and therefore the cat possibly experiencing greater anxiety or perceived conflict [e.g., (53)] in this condition. This interpretation is plausible, given the higher frequency of other, more direct, cat conflict-linked measures identified in this condition. Interestingly, frequencies of the cat directly approaching and making contact with the person did not differ significantly between conditions. This potentially suggests that whilst the cat's comfort was negatively impacted by the participants' style of handling in the control (perhaps motivating them to periodically put more distance between themselves and the person), these potentially negative experiences did not affect the cats' general intent to physically/socially engage with the person overall.

Interestingly, applying the guidelines did not significantly positively impact participants' impressions of cats or their “desirability.” This is surprising, given that human-directed affiliative behaviours (54, 55) and aggression (14, 16) are typically considered desirable and undesirable respectively. However, the guidelines had no significant *negative* impact on participants' impressions of cats either. This would suggest that prioritising cats' comfort and human safety, by encouraging “best practice” approaches during HCI, can potentially be achieved without limiting humans' ability to form positive associations with cats. As such, it is unlikely that applying the CAT guidelines would negatively impact upon rehoming rates, and might actually lead to increased adoption [e.g., (54, 55)], although this hypothesis requires further testing.

What is potentially concerning, is that whilst participants' ratings of cats' level of friendliness and degree of comfort did not differ between conditions, objective measures of cats' behaviour suggested their comfort during the control condition was compromised. This may indicate that participants were not sensitive to the degrees to which behaviours associated with positive/negative valence were present/absent in the cats they interacted with, or that they were at least unaware of the relevance of these behaviours to the cats' comfort. Such interpretations would appear congruent with other findings suggesting humans tend to struggle to correctly differentiate between positive and negative affective states in cats, based on their behavioural expressions (56), and do not focus on these cues when making adoption decisions (55). However, absolute occurrences of conflict and agonistic behaviours were relatively low across both conditions (meaning they could be easily missed by participants), and affiliative behaviours comparatively more frequent. Additionally, cats' control and intervention ratings were completed by different people, rather than by the same person making a comparative between-condition judgement of the same cat. Both of these factors could equally explain the lack of significant between-condition differences concerning the “friendliness” and “comfort” ratings given to cats. Future educational interventions aimed at increasing humans' awareness of the important (but less overt) behavioural signs of cat comfort/discomfort during HCI would be beneficial none-the-less.

Effect sizes for the differences in cats' behaviour between the control and intervention were generally moderate. However, the protocols put in place to protect cats' well-being and humans' safety during the study are likely to have mitigated the negative impact of the control condition on cats' experiences during HCI to a degree. For example, whilst the “control” condition was intended to encourage more “naturalistic” styles of HCI, the reality of instructing participants to remain seated and to not follow the cat, pick them up, or disturb them whilst hiding, meant the control already incorporated several key elements of “best practice” handling. Additionally, by only including cats considered well-socialised toward people, and subsequently removing any cats that showed more intense aggressive responses during the study (i.e., potential to cause real harm to participants), it is very likely that we selected against the cats that might actually have benefitted most from the CAT approach to HCI. This likely explains the relatively low levels of human-directed aggression and conflict-based behaviours across all observations, as well as the lack of effect of cats' temperament on relative differences in the occurrence of “positive” and “negative” behavioural responses between conditions. Indeed, mode L-CAT scores for the traits “Friendliness” and “Frustration” were near the maximum and minimum end of the scale, respectively, suggesting this was a relatively homogenous population of well-socialised cats, with minimal tendencies toward human-directed aggression. It is therefore anticipated that application of the CAT guidelines within more typical cat shelter populations (i.e., those that are less friendly but more anxious or easily frustrated), and when contrasted against more usual baseline styles of human handling, would produce even greater positive effects on cats' behaviour.

In addition to their application amongst members of the public visiting the cattery, the CAT guidelines may be particularly useful when incorporated into the standard HCI practices occurring between cats and their caretakers. As residing within the cattery environment is typically a stressful experience for cats, handling and husbandry protocols that promote positive cat well-being are essential (57). Several studies have investigated the potential benefits of exposing cats within a rehoming environment to “gentling” programmes (i.e., a human stroking and vocalising to a cat) in order to promote relaxation and improve well-being (19, 58). However, specific methods of “gentling,” as described in these studies, were mostly unclear and/or implied that for at least some cats (i.e., those behaving aggressively), individuals were not provided with choice and control over the nature of the HCI (19). Indeed, within this latter study, certain individuals responded fearfully and/or defensively to “gentling” and did not appear to benefit from this form of HCI in the same way as did cats that responded in a positive, affiliative manner (19). These approaches to HCIs may therefore be beneficial for some, but not all cats, and in certain cases may induce (or at least exacerbate) negative affective states. Adopting HCI methods (such as the “CAT”) which allow cats to either “opt in” or “opt out” of HCIs, as well as dictate their nature, are likely to ensure HCIs have positive impacts on cats. Such approaches may also ensure that HCIs do not inadvertently induce experiences of stimulus flooding or subsequent “learned helplessness” (59, 60) that could otherwise arise due to the cats' lack of perceived control or ability to remove themselves from the (potentially) aversive situation they are being exposed to [e.g., (61)].

Human-directed aggression is typically considered a “problem behavior” requiring professional intervention (62). Its presence may negatively impact cats' well-being, in addition to the cat-owner relationship (63), potentially influencing cat relinquishment decisions (64, 65). Application of the CAT approach to HCIs within the home may therefore help to promote more positive cat well-being and cat-human relationships, and reduce the likelihood of owners surrendering their cats. Whilst little information exists to enable accurate quantification of the numbers of cats involved in AAIs, the current popularity of AAIs within both educational and therapeutic settings [e.g., (1, 2, 34)] suggests this may be considerable. With greater inclusion of cats within AAI programmes, and increased popularity of Cat Cafes (31), comes a greater risk of cats exposed to suboptimal HCI, leading to human injury and cat discomfort. As the principles of the “CAT” are suitably generic for broad application, and associated training materials (see Supplementary Files 3–6) easily modifiable, effective application of the CAT guidelines within both the domestic home and a range of other HAI contexts are anticipated. Further studies to test “CAT” efficacy within such situations are therefore recommended.

Inter-reliability for the measures analysed within this study were established via coding contributions from a second coder that was blind to the conditions within observations. However, due to practical limitations, the majority of data used in the main analysis were coded by a (technically) un-blind individual, potential creating a source of coder bias. Therefore, where CAT efficacy testing is undertaken in further studies, suitable experimental protocols should be utilised to ensure that all video observations can be coded in a fully blind manner. Additionally, while highly reliable between coders, ratings of participants' handling styles were relatively subjective. Thus, further, more objective investigations of the impact of the CAT intervention on humans' behavioural styles during HCI are recommended. These may help to better understand the specific differences in humans' behaviour that underpin the more positive behavioural responses observed in cats following the intervention.

## Data Availability Statement

The data presented in the study are deposited in the Figshare repository as ‘Supplementary Data S2' and can be accessed here: https://figshare.com/articles/dataset/Supplememntary_data_S1-8/14828397.

## Ethics Statement

Ethical approval for the study was granted by the delegated authority of Nottingham Trent University, Research Ethics Committee ref: ARE192011. All aspects of experiments were performed in accordance with relevant guidelines and regulations. All personal data provided by participants for the purposes of the study were stored in line with current GDPR guidelines. All cats were periodically monitored during data collection by cat welfare experts LF, RF-W, CH and JP, in addition to the wider cattery team. Any cats observed showing signs of distress, illness or the potential to injure the participant were immediately removed from the study and human participants were free to leave the experiments at any time.

## Author Contributions

LF conceived and designed the project, collected data, performed the data analysis, and wrote the manuscript. LR coded the videos and performed the data extraction and analysis. RF-W, CH, and JP collected the data. CH also coded the videos. All authors approved the submitted version of the article.

## Conflict of Interest

The authors declare that the research was conducted in the absence of any commercial or financial relationships that could be construed as a potential conflict of interest.

## Publisher's Note

All claims expressed in this article are solely those of the authors and do not necessarily represent those of their affiliated organizations, or those of the publisher, the editors and the reviewers. Any product that may be evaluated in this article, or claim that may be made by its manufacturer, is not guaranteed or endorsed by the publisher.

## Supplementary Material

All supplementary materials are available via 10.6084/m9.figshare.14828397.
